# Short Face Correction with Orthognathic Surgery Using an Autologous Mandibular Angle Graft

**DOI:** 10.1007/s12663-025-02620-2

**Published:** 2025-06-28

**Authors:** Martin Orozco-Fernández, Alejandra Rojas-Ponte, Maria Clara Jaramillo, Juan Pablo López

**Affiliations:** 1 Chief Department Oral and Maxillofacial Surgery Hospital, Universitario Clínica Colombia, Bogotá, Colombia; 2 Maxillofacial Surgery Program, Universidad El, Bogotá, Colombia; 3 Department Oral and Maxillofacial Surgery Hospital, Universitario Clínica Colombia, Bogotá, Colombia; 4 Department Oral, Maxillofacial Surgery Clinica Reina Sofía, Bogotá, Colombia; 5 Oral and Maxillofacial Surgeon, Hospital Universitario Fundación Santa Fe de Bogotá, Bogotá, Colombia; 6 Universidad El Bosque, Clinical Oral Epidemiology Research Unit, UNIECLO, Bogotá, Colombia

**Keywords:** Orthognathic surgery, Osteotomy, Bone transplantation

## Abstract

The short face has been challenging in orthodontic and surgical management due to unfavorable aesthetic results. More surgical alternatives should be described in the literature. The objective of this report is to propose a novel surgical technique complementary to orthognathic surgery for the management of the short face. To this end, a retrospective series of 10 cases analyzes and describes a novel method using autologous mandibular angle grafts after mandibular angle recontouring. The autologous mandibular angle graft technique is a simple, minimally invasive technique that allows mandibular angle ostectomies as interposition grafts for chin and maxilla descents, improving aesthetic results and bone stability.

## Introduction

The short face dentofacial deformity (DFD) is an alteration of facial harmony caused by a discrepancy between the upper and lower facial heights. This alteration has a vertical and horizontal commitment that generates sad or aged faces. Its mandible is characterized by a very horizontal mandibular plane and prominent mandibular angles [1]. Short faces have been classified according to the location of the problem: maxillary vertical defect or mandibular posterior vertical deficiency. The classification is based on multiple cephalometric studies that helped make surgical decisions [2].

The short face corresponds to the least common dentofacial deformity, so few patients undergo orthognathic surgery to achieve its correction. This makes evaluating aesthetic results with different surgical techniques even more difficult. Additionally, plane changes and vertical corrections usually require the placement of bone grafts that facilitate healing and promote the stability of the movement performed, which generates additional costs [1].

The present study aims to propose a simple technique complementary to bimaxillary orthognathic surgery to correct aesthetic defects in the short face. This technique uses the bone segments resected during mandibular angle plasty as an autologous interposition graft, improving aesthetic and stability.

## Materials and Methods

This retrospective case series analyzes a novel technique using autologous mandibular angle grafts as a complementary procedure to bimaxillary orthognathic surgery. The consecutive number of patients with dentofacial anomalies from the Clinica Universitaria Colombia, Bogotá, DC, Colombia, was analyzed. A search of the operated cases was conducted, and class II patients with short faces with preoperative and postoperative studies were selected. Exclusion criteria were class II patients with short faces where techniques other than those described for vertical correction were used. After analyzing the registered cases, the following ten cases were selected (Table 1)**.**

**Table 1:** Summary of demographic and cephalometric data

Case	Age	Gender	Movement direction	ENA mm	A mm	B mm	Pog mm	Me mm	Superior incisor mm
Case 1	20 years	Female	Anterior	2.9	3	7.5	6.9	6.4	2.9
			Down	3	3	7.4	11.7	11.3	3
Case 2	33 years	Male	Anterior	4	4	8.5	13.7	13.4	4
			Down	0	0	3.9	7.6	7.4	0
Case 3	35 years	Female	Anterior	4.0	4.0	8.42	14.35	14.31	4.00
			Down	1.04	1.08	1.65	3.11	2.97	2.97
Case 4	39 years	Female	Anterior	6.00	6.0	3.31	0.34	0.19	6.00
			Down	2.00	2.00	2.33	2.98	2.89	2.00
Case 5	40 years	Male	Anterior	4.39	4.84	10.17	14.26	14.29	6.68
			Down	0.39	0.01	4.55	9.07	9.09	0.01
Case 6	25 years	Male	Anterior	5.00	5.00	7.14	6.25	5.05	5.00
			Down	4.04	4.01	5.99	11.01	10.01	3.87
Case 7	39 years	Male	Anterior	4.99	4.99	10.63	12.03	11.68	5.07
			Down	0.00	0.00	1.93	5.98	5.81	0.00
Case 8	33 years	Male	Anterior	3.27	3.68	7.87	11.81	11.77	5.66
			Down	0.00	0.17	3.88	6.19	6.16	0.07
Case 9	27 years	Female	Anterior	4.01	3.99	11.71	17.32	16.72	3.93
			Down	1.07	1.04	4.09	7.60	6.74	0.92
Case 10	24 years	Male	Anterior	8.90	8.52	6.04	2.56	3.13	7.22
			Down	3.55	3.30	3.30	4.12	3.36	3.35
Case 11	42 years	Female	Anterior	4.8	5.29	11.84	18.01	18.08	7.17
			Down	0.3	0.04	3.07	5.09	5.14	0

### Case 1

A 20-year-old female patient was referred from the orthodontic service to the oral and maxillofacial surgery service to evaluate dentofacial anomalies. She had a history of unspecified respiratory allergies without chronic treatment. The patient’s consultation was to improve her bite and her facial appearance. Clinical examination shows a class II patient with reduced lower facial height, a horizontal mandibular plane, and marked mandibular angles. Intraorally, a class II malocclusion is observed with an increased vertical overbite where the upper incisors cover the lower incisors by approximately 90%.

**Fig. 1:**
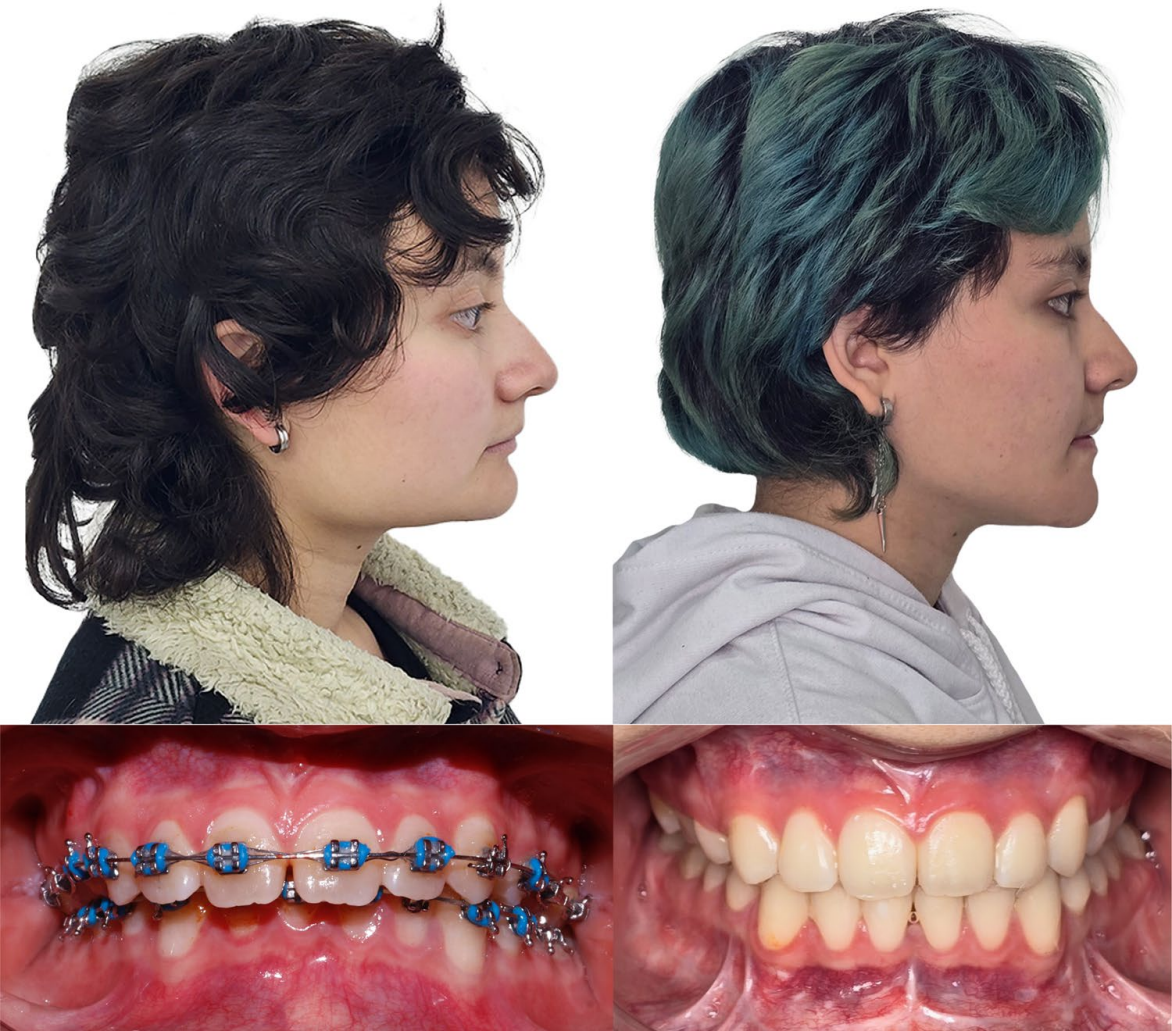
Pre- and postoperative profile images in case 1 showing the difference in facial height and mandibular projection, and intraoral front view

**Fig. 2:**
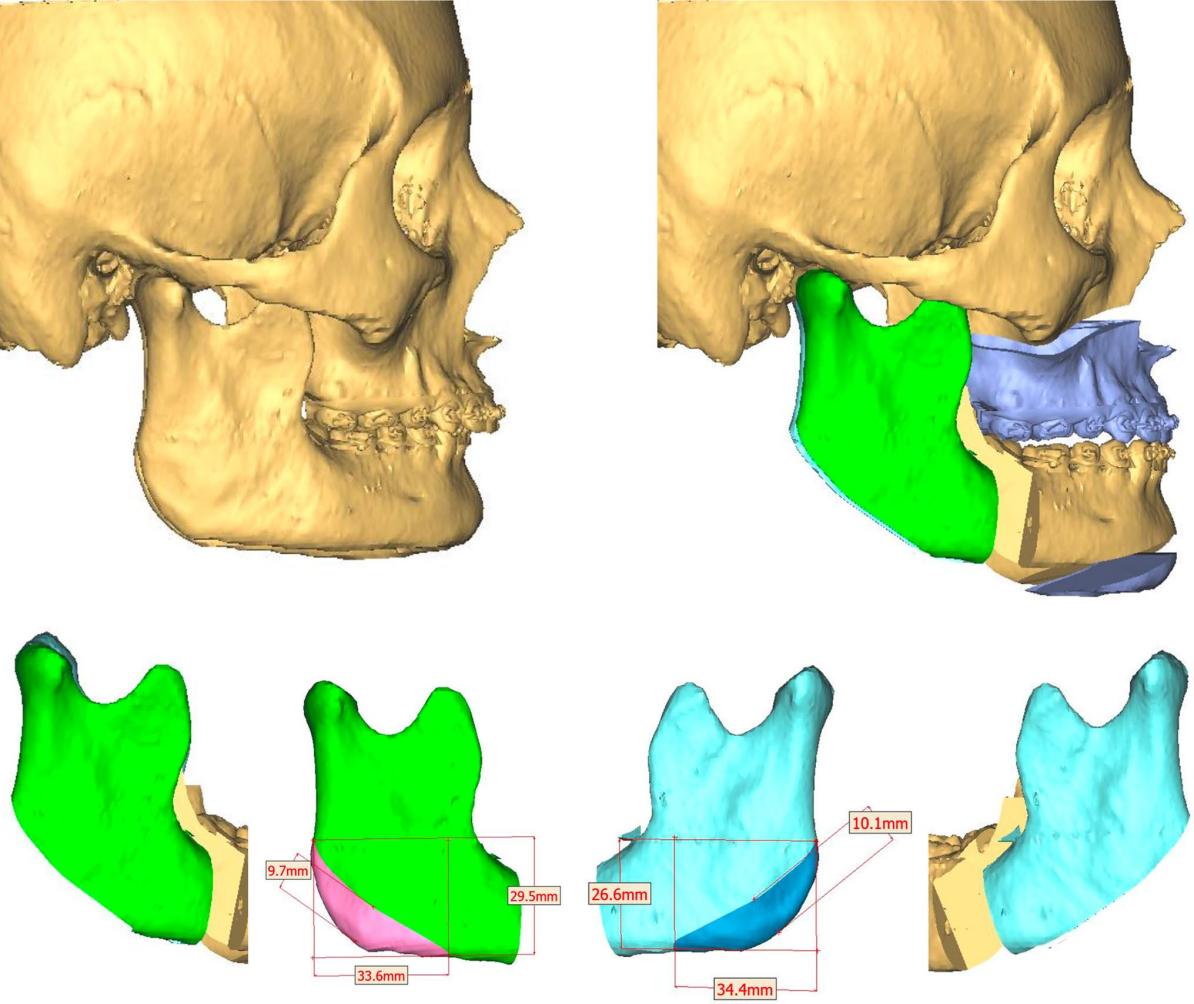
Preoperative virtual planning in case 1 showing ostectomies design and graft planning

Clinical findings are confirmed during virtual planning with computed tomography, and the treatment plan is defined. This is bimaxillary orthognathic surgery with a maxillary first sequence, where advancement and descent of 3 mm and a clockwise yaw correction of 1 degree are planned. The mandible is brought into occlusion with advancement and alignment. Concerning the chin, advancement, and descent of 5 mm are made. Currently, the patient has been under follow-up for 22 months without complications (Figs. 1, 2)**.**

### Case 2

A 33-year-old male patient arrives at the oral and maxillofacial surgery service for evaluation due to the sensation of a small face. He had a history of high blood pressure under study but without necessary medication. On physical examination, the patient presents a reduced lower facial height, a horizontal mandibular plane, and marked mandibular angles. Intraorally, a class II malocclusion is observed with an increased vertical overbite where the upper incisors cover the lower incisors by approximately 80%, the upper and lower dental midline coinciding.

**Fig. 3:**
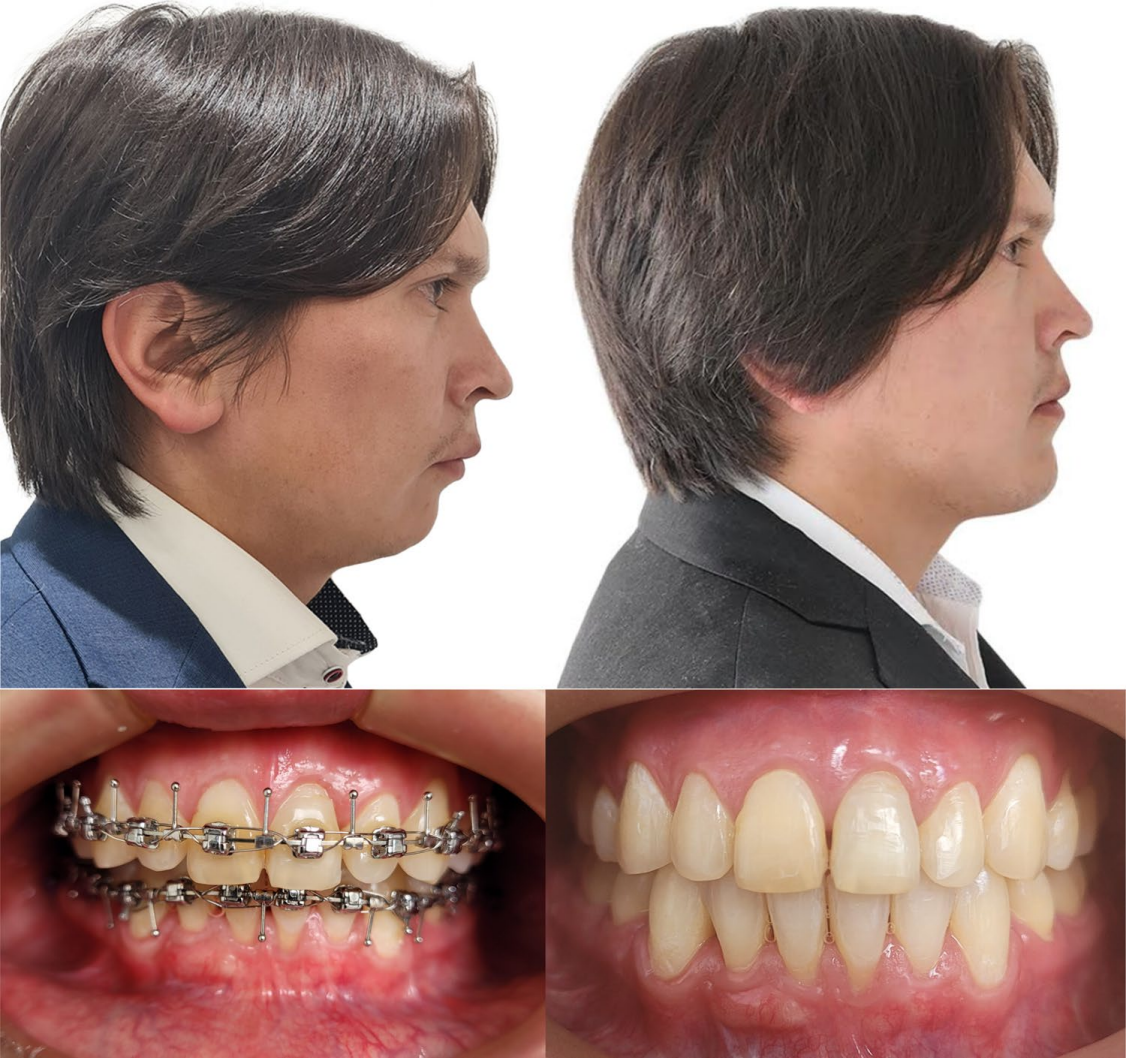
Pre- and postoperative profile image in case 2 showing the difference in facial height and mandibular projection, and intraoral front view

**Fig. 4:**
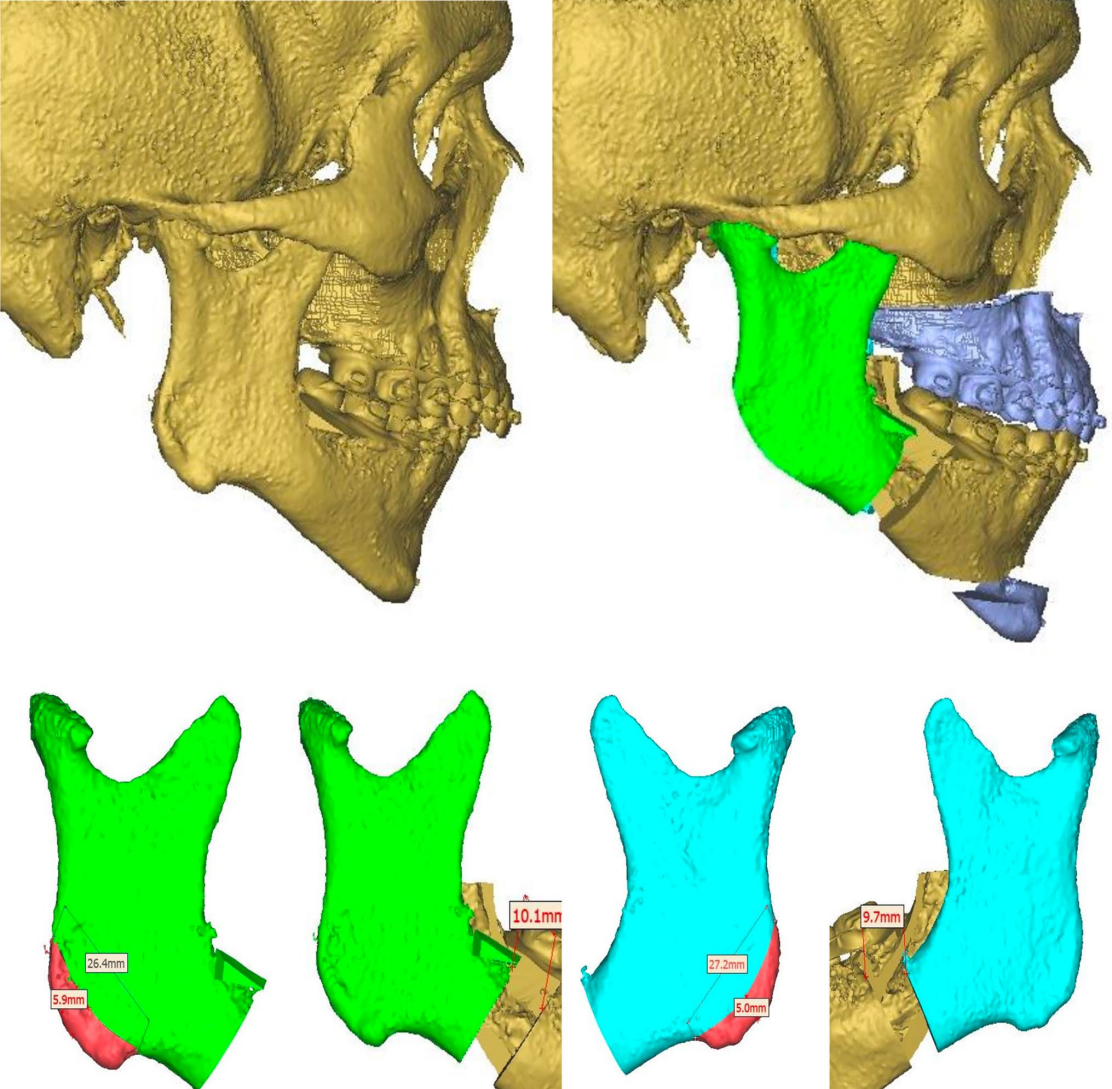
Preoperative virtual planning in case 2 showing ostectomies design and graft planning

Clinical findings are confirmed during virtual planning with computed tomography, and the treatment plan is defined. The mandibular sequence starts with a bilateral advancement of 9 mm, followed by a LeFort I osteotomy of 4 mm advancement, and finally, mentoplasty of 6 mm advancement and 3 mm descent. Approximately 5 mm of bilateral gonial angles were resected for the mentoplasty space. Currently, the patient has been under follow-up for 34 months without complications (Figs. 3, 4).

### Surgical Technique

Bimaxillary orthognathic surgery was performed with a conventional high LeFort I technique and bilateral sagittal osteotomy of the Hunsuck type of mandible. In the case where a maxilla-first sequence is performed, the approach is not sutured once the fixation of the osteosynthesis material is finished. A bilateral sagittal osteotomy is then performed using the technique described previously to mobilize the proximal segment and easily take the graft from the mandibular angles. A dislocation maneuver of the proximal segment is performed for better visualization, and the cut is made with piezosurgery-type ultrasonic cutting devices using an angled tip. These grafts are preserved in a container with Ringer’s lactate, while the mandibular surgery is completed and the chin osteotomy is performed. In mandible-first sequence cases, the procedure was similar. Sagittal osteotomy is performed to more easily obtain the mandibular angle segments by mobilizing the proximal segment. The grafts are left in Ringer’s lactate to be then placed in the maxilla and chin.

**Fig. 5:**
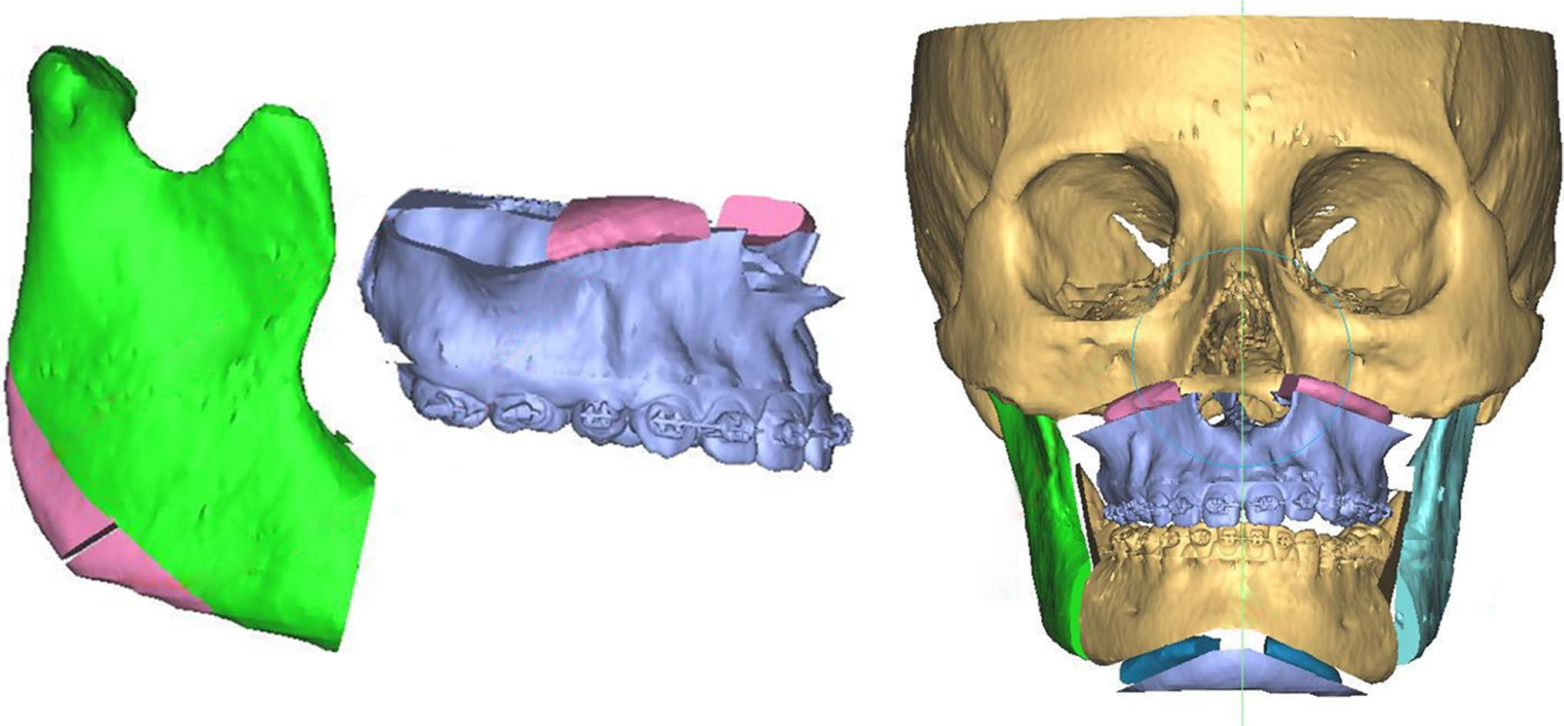
Virtual planning of bilateral angular cut and interposition bone graft

Considering the bone spaces in osteotomies to recover the lower anterior facial height, a mandibular angle ostectomy is virtually planned to profile and improve the appearance of the posterior facial height. The resected bone segment on each side is divided into two symmetrical segments of approximately 9 mm for case #1 and 5 mm for case #2. The four bone segments obtained are used as autologous grafts for interposition. They are used as follows: One on each side in the space created during the descent of the jaw. The other two bone segments are used in the space created by the descent of the chin. Autologous bone grafts promote bone healing more quickly due to contact and favor the stabilization of the mobile jaws, reducing the possibility of recurrence and bone nonunion. Both cases have a satisfactory aesthetic, functional, and occlusal result. All patients are asymptomatic, and none have presented complications (Fig. 5).

**Fig. 6:**
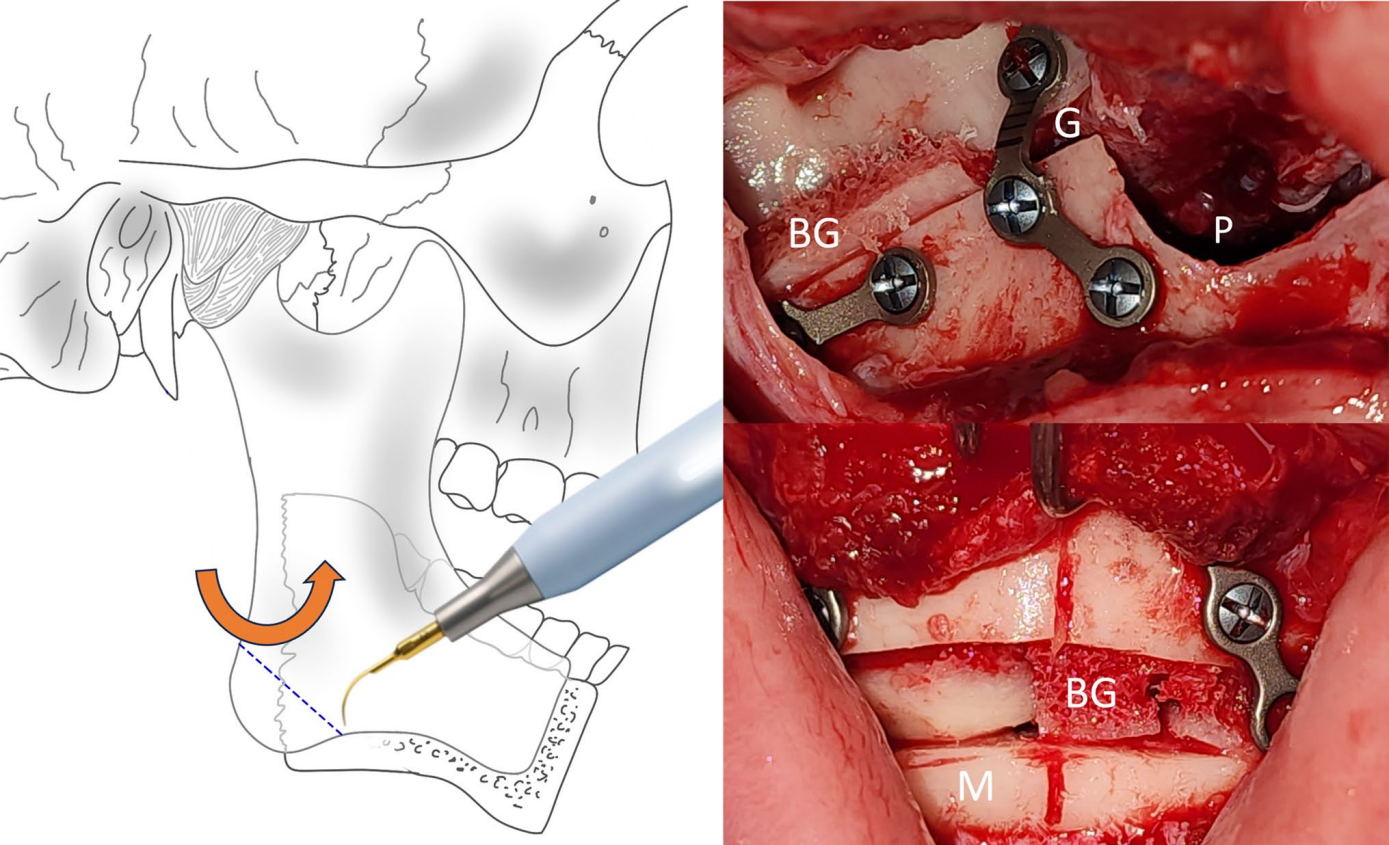
*Left* Diagram of how to cut the goniac angle with a piezoelectric device. *Right* Intraoperative view of LeFort I and menton osteotomy with bone grafts between the bone gaps. *Abbreviations* G, gap; M, menton; P, pyriform; BG, bone graft

The indication for mandibular angle grafts in patients with a short face depends on the exaggerated size of the gonial angle and the necessary recontouring. Depending on the resected area, these grafts are placed in the bone gap with or without additional grafts (Fig. 6). With the help of virtual planning, it is possible to determine the necessary amount of resection (bone volume of the graft) and the planned gap.

## Discussion

The short face corresponds to the least common dentofacial deformity, which makes aesthetic evaluation with different surgical techniques difficult. Likewise, few treatment options are described that address the problem comprehensively. Bone grafts are typically necessary for plane changes and vertical corrections to enable proper healing and movement stability, resulting in extra expenses.

The management of vertical maxillary deficiency has traditionally been handled with maxillary descents. Its main problem was compromised stability due to the lack of bone contact. In 1974, Hogeman introduced the possibility of performing LeFort I osteotomy with interposition grafts to mitigate these stability problems. This was further improved with rigid fixation in the maxilla [3].

Another factor to consider in orthognathic and orthodontic surgery management is the curve of Spee. A clinical imaging study studied the relationship of the curve of Spee with the short face. They suggest a crucial aesthetic benefit in maintaining a pronounced curve of Spee because it is associated with an increase in the height of the soft tissue and its relationship with the lower lip. All this is based on an intuitive geometric principle [4]. In the cases described in this article, moderately pronounced curves of Spee were taken into account.

Bilateral masseteric hypertrophy due to overactivity can lead to an enlargement of the bony mandibular angle. The horizontalization of the mandibular plane can affect the mandibular lines and alter facial aesthetics [5]. In the two previous cases, botulinum toxin was not used in the masticatory muscles, considering that the mandibular angle osteotomy creates space for the masseter muscles and does not interfere with bone movements. However, in cases of significant advancements, muscle hypertrophy, or counterclockwise rotation, it can be used both in the masseter muscles and in the anterior belly of the digastric muscle. Botulinum toxin, a vial of 100 IU 1 month before surgery could be performed. Myotomy is an invasive and painful technique; therefore, the technique described in this article offers an alternative management approach.

A recently published case demonstrates a class II patient with a short face and limited oral opening. The author performed vertical extraoral osteotomies, myotomy, coronoidectomy, and mentoplasty. This procedure was satisfactory for the patient because the aesthetic, functional, and occlusal objectives were met [6]. It is an exciting case with good results. On the other hand, management with botulinum toxin in the masseter muscle and intraoral orthognathic surgery could also be an alternative. Unlike the currently reported case, no extraoral incisions were made, and a maxillary descent was used to achieve a better aesthetic result at lower anterior facial height.

The present study proposes a simple technique complementary to bimaxillary orthognathic surgery for correcting aesthetic defects in the short face. The method describes bone segments resected during intraoral plasty of the mandibular angle’s characteristic of this dentofacial deformity. These autologous grafts allow for a simple, economical, and easy-to-perform technique that improves aesthetic and functional results.
